# Supplementary material for: Predicting visual function by interpreting a neuronal wiring diagram
Source: Nature. 2024 Oct 2;634(8032):113–23. doi: 10.1038/s41586-024-07953-5 (PMC11446822; doi:10.1038/s41586-024-07953-5)
Supplement: Supplementary file 6 — CRF and ERF predictions for individual TmY4 and TmY9 cells. Analogous to Supplementary Data 3, but for TmY target types. Shown are the top four monosynaptic pathways, the strongest pathway passing through each of the top ten intermediary types (ranking from Extended Data Fig. 7), and the trisynaptic pathway Tm1–TmY–Dm3–TmY (see the section entitled Prediction of spatial normalization). [file 41586_2024_7953_MOESM6_ESM.zip › DataS4/TmY9qΓèÑ/720575940620707719.pdf]

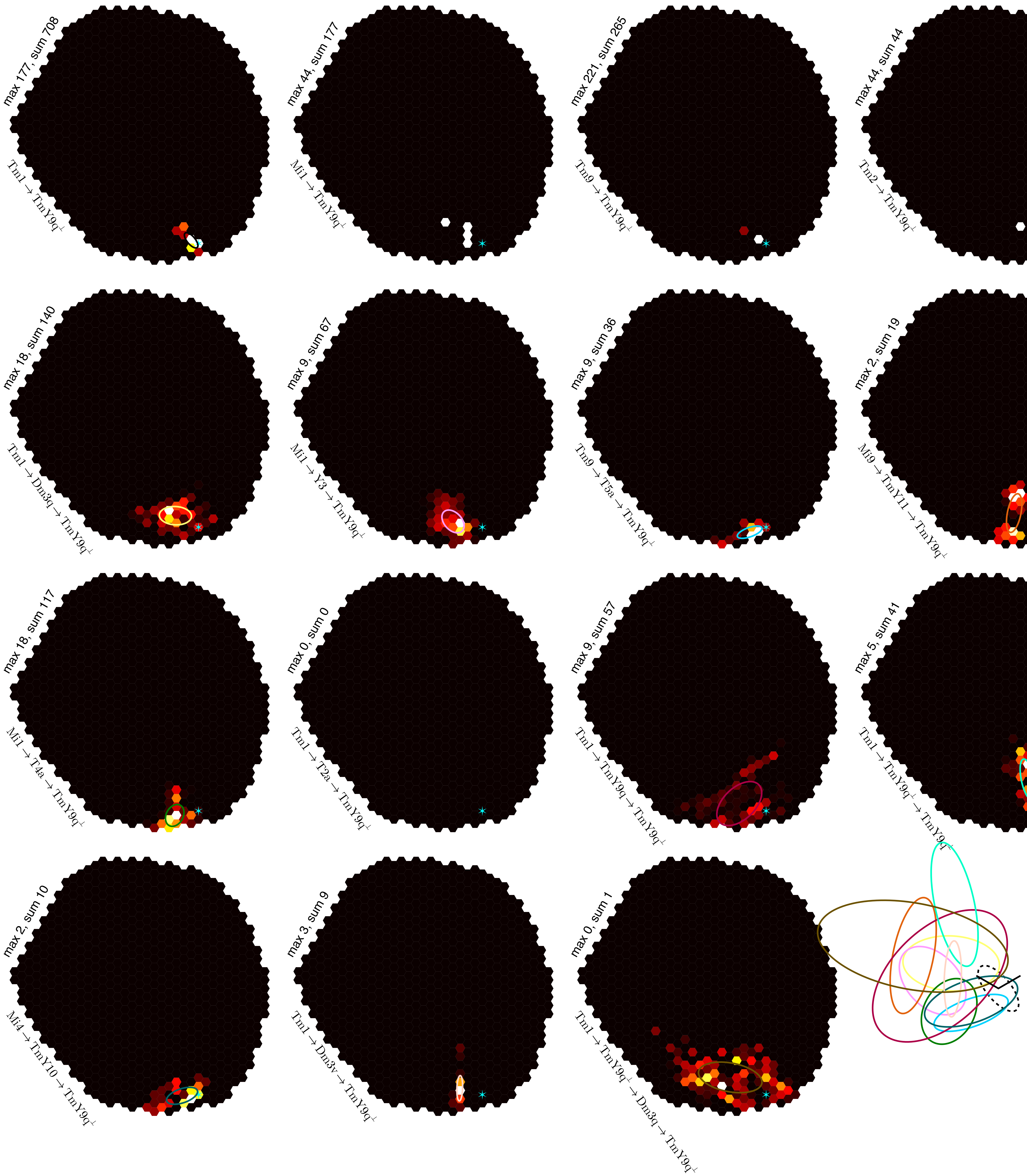
max 177, sum 708
Tm1 → TmY9q⊥
max 44, sum 177
Mi1 → TmY9q⊥
max 221, sum 265
Tm9 → TmY9q⊥
max 44, sum 44
Tm2 → TmY9q⊥
max 18, sum 140
Tm1 → Dm3q → TmY9q⊥
max 9, sum 67
Mi1 → Y3 → TmY9q⊥
max 9, sum 36
Tm9 → T5a → TmY9q⊥
max 2, sum 19
Mi9 → TmY11 → TmY9q⊥
max 18, sum 117
Mi1 → T4a → TmY9q⊥
max 0, sum 0
Tm1 → T2a → TmY9q⊥
max 9, sum 57
Tm1 → TmY9q → TmY9q⊥
max 5, sum 41
Tm1 → TmY9q⊥ → TmY9q⊥
max 2, sum 10
Mi4 → TmY10 → TmY9q⊥
max 3, sum 9
Tm1 → Dm3v → TmY9q⊥
max 0, sum 1
Tm1 → TmY9q⊥ → Dm3q → TmY9q⊥
